# Short-term Outcomes of Collagen Crosslinking for Early Keratoconus

**Published:** 2011-07

**Authors:** Akbar Derakhshan, Javad Heravian Shandiz, Masumeh Ahadi, Ramin Daneshvar, Habibollah Esmaily

**Affiliations:** Mashhad University of Medical Sciences, Mashhad, Iran

**Keywords:** Cornea, Keratoconus, Riboflavin, UVA Radiation, Collagen Crosslinking

## Abstract

**Purpose:**

To assess the efficacy of collagen crosslinking with riboflavin and ultraviolet A (UVA) radiation for treatment of early keratoconus.

**Methods:**

Thirty-one eyes of 22 patients with early keratoconus were included in this study. All patients underwent slit lamp examination and assessment of uncorrected visual acuity (UCVA), best spectacle-corrected visual acuity (BSCVA), intraocular pressure, corneal topography and pachymetry. Collagen crosslinking was performed without epithelial removal. Riboflavin was applied to the cornea every 3 minutes 30 minutes before UVA irradiation and every 5 minutes thereafter. Patients were re-assessed 1, 3, and 6 months after treatment.

**Results:**

Postoperatively, UCVA increased by 2 Snellen lines and BSCVA was improved by 1.7 Snellen lines (P < 0.001). Spherical equivalent refractive error was reduced by 0.55 D, and maximum and mean K values were decreased by 0.65 D and 0.51 D respectively (P < 0.05 for all comparisons). Evidence of regression was present in 71% of treated eyes.

**Conclusion:**

Collagen crosslinking demonstrated significant improvement in vision with reduction in corneal power and spherical equivalent refractive error in eyes with early keratoconus.

## INTRODUCTION

Keratoconus is a degenerative non-inflammatory disease of the cornea, resulting in distortion, apical thinning and central scarring. These corneal changes lead to decreased vision due to high irregular astigmatism and less frequently, central corneal scarring. The condition usually begins at puberty and tends to progress during adolescense.^1^,^2^ Treatment consists of glasses, rigid contact lenses and intracorneal rings early in the disease, however none of these modalities affect progression of the condition. Eventually, penetrating keratoplasty may be required in advanced cases to restore vision.^3^,^4^

Collagen crosslinking has been studied during recent years by Wollensak and colleagues^4^–^9^, as well as others^10^–^14^. Collagen crosslinking, using riboflavin and ultraviolet A (UVA) light, has been shown to alter the biomechanical, thermomechanical, and morphological properties of the cornea. It increases corneal rigidity by almost 300% and enhances its resistance to proteolytic enzymes.^7^,^15^–^17^ Numerous clinical studies on collagen crosslinking in progressive keratoconus have shown an arrest in progression and even regression in the majority of patients.^4^,^12^–^14^,^18^,^19^ Longitudinal studies have demonstrated visual improvement and long-term stable outcomes after collagen crosslinking.^20^ The principal goals of such therapy are to increase corneal rigidity, stabilize its refractive and biomechanical properties and thus improve vision.

The current study was designed to evaluate the effectiveness of this procedure as primary treatment for eyes with early keratoconus.

## METHODS

This study was performed at Khatam-al-Anbia Hospital affiliated to Mashhad University of Medical Sciences. Informed written consent was obtained from each patient before participation. Beginning in January 2008, 31 eyes of 22 patients with early keratoconus were enrolled for collagen crosslinking. The diagnosis was made based on clinical examination and videokeratographic findings. Preoperative evaluations included measurement of uncorrected visual acuity (UCVA), best spectacle-corrected visual acuity (BSCVA), intraocular pressure (IOP), corneal computerized topography (Technomed, Baseweile, Germany), ultrasonic pachymetry (Tomey, Erlangen, Germany), and slit lamp and fundus examinations. All assessments were repeated one, 3, and 6 months after collagen crosslinking. Exclusion criteria consisted of corneal thickness less than 400 μm, signs of advanced keratoconus (Vogt’s striae or corneal scarring), history of herpetic keratitis, and concurrent infectious or autoimmune disease.

Since all patients had early keratoconus without corneal scarring, we preferred a minimally invasive procedure omitting epithelial removal which has been shown to be equally safe and effective as with epithelial removal.^14^,^21^ Treatment was performed under sterile conditions using topical anesthesia. After inserting a wire lid speculum, the photosensitizing solution (0.1% riboflavin in 20% dextran) was instilled every 3 minutes for 30 minutes. Before UVA exposure, penetration of riboflavin into the anterior chamber was confirmed by slit lamp examination. A double UVA diode was then placed 5 cm from the cornea and UVA light irradiated the central cornea at 370 nm and with radiance of 3 mW/cm^2^. During exposure, the riboflavin solution was applied to the cornea every 4 to 5 minutes to ensure continuous saturation; balanced salt solution was instilled on every 2 minutes to prevent dryness and dehydration. After crosslinking, a topical antibiotic was prescribed for 5 days.

## RESULTS

**Figure 1:**
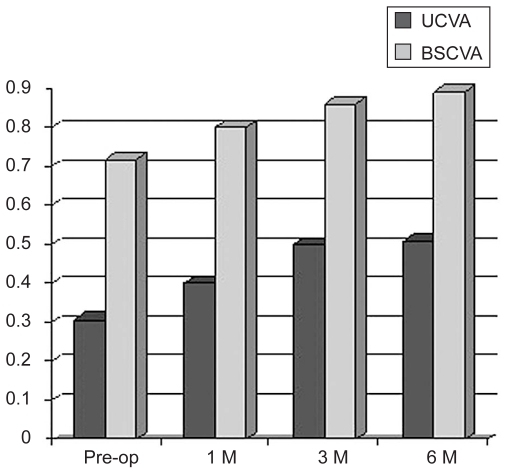
Changes in Snellen visual acuity.

Thirty-one eyes of 22 patients including 11 male and 11 female subjects with mean age of 22.3 ± 6.8 (range, 15 to 44) years, underwent collagen crosslinking for early keratoconus. Mean Snellen UCVA was 0.31 ± 0.21 preoperatively and 0.51 ± 0.27 postoperatively. Mean Snellen BSCVA was 0.72 ± 0.18 preoperatively and 0.89 ± 0.20 postoperatively (Fig. 1). Comparison of preoperative and 6-month follow-up data showed an increase of 2.0 ± 1.8 lines in UCVA (P < 0.001) and 1.7 ± 1.1 lines in BSCVA (P < 0.001). UCVA and BSCVA improved by more than one Snellen line in 27 (87%) eyes and remained unchanged in four eyes (13%).

**Figure 2:**
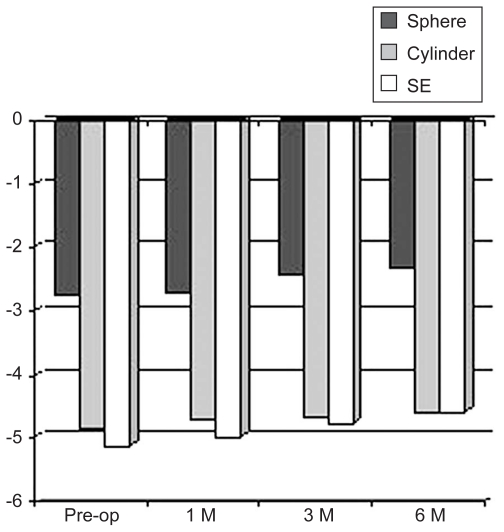
Changes in refraction (diopters). SE, spherical equivalent

Mean spherical equivalent refractive error was −5.13 ± 3.67 D preoperatively and −4.58 ± 3.27 D postoperatively (Fig. 2). Mean reduction in spherical equivalent was 0.55 ± 0.92 D (P = 0.004) over the follow-up period in 22 eyes (71%).

**Figure 3:**
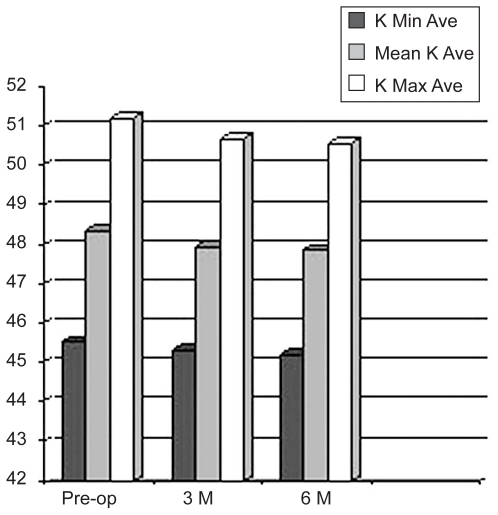
Changes in minimum, maximum, and mean K values (D). K Min Ave, average minimum K value; Mean K Ave, average mean K value; K Max Ave, average maximum K value

Preoperatively, maximum and mean keratometric (K) values were 51.21 ± 4.97 D and 48.38 ± 4.24 D respectively. Postoperatively, these values were decreased to 50.56 ± 3.87 D and 47.87 ± 3.85 D respectively (Fig. 3). An average decrease of 0.65 ± 1.26 D in maximum K value (P = 0.007) and 0.51 ± 0.93 D in mean K value (P = 0.005) was observed in 24 eyes (77%) but the indices remained unchanged in four eyes (13%). These results were observed at three months and remained stable up to 6 months. We observed an increase of 0.12 ± 0.21 D in maximum K value (P = 0.17) and 0.10 ± 0.07 D in average K value (P = 0.13) in 4 eyes (10%).

In this study, 13 patients were treated unilaterally. In 3 of these subjects, the untreated fellow eye demonstrated signs of advanced keratoconus including one person who had previously undergone penetrating keratoplasty; 4 patients had unilateral keratoconus (the fellow eye showed no evidence of ectasia at baseline or on subsequent examinations); and the remaining 6 individuals showed signs of forme frust keratoconus in the contralateral eye. In the latter subset of patients four eyes (66.6%) demonstrated an average increase of 0.61 D and 0.46 D in maximum and mean K values after 6 months, respectively.

Mean central corneal thickness (CCT) was 485 ± 29.6 μm before treatment and 494 ± 30.8 μm thereafter; CCT increased by an average of 9.1 ± 11.2 μm (P < 0.001).

Mean IOP was 13.5 ± 2.1 mmHg pre- and 13.6 ± 2.2 mmHg postoperatively (P = 0.565). During follow-up, corneal and lenticular transparency remained unchanged. Moreover, no side effects such as persistent epithelial defects or cataracts were encountered.

## DISCUSSION

Our study was performed on patients with early keratoconus and no signs of advanced disease, such as Vogt’s striae and corneal scarring. The results showed an increase in vision together with a decrease in refractive error and corneal curvature.

Vision improved in most patients; this improvement began from the first month, slightly increased by the third month and remained stable up to six months. Visual improvement could have been due to the decrease in refractive error, corneal steepness and astigmatism. In some cases, improvement in UCVA and BSCVA occurred before corneal changes were detectable. This may have been related to changes in refractive index in some areas of the cornea and formation of a multifocal cornea.

We observed a statistically significant increase in CCT, the clinical significance of which remains to be ascertained. Following crosslinking, structural corneal changes alter the velocity of ultrasound which may influence pachymetric findings.^20^ Another possible explanation is that enhanced biomechanical rigidity in the cornea may change the pattern and distribution of collagen fibers and actually increase CCT.^7^,^14^ Due to the small number of patients and limited follow-up, further studies are required to elucidate this matter.

No significant change was observed in IOP as measured by Goldmann applanation tonometry. We suggest that corneal rigidity and its effects on IOP be investigated in future studies using the ocular response analyzer (ORA).

Topographic findings demonstrated mean reduction of 0.65 D in maximum K and 0.51 D in average K values in 77% of our patients. Wollensak et al^4^ reported mean reduction of 2.01 D in maximum K after 3 years, and Caporossi et al^13^ reported mean reduction of 2.10 D in maximum K values after 6 months. Raiskup-Wolf et al^20^ described reduced curvature of 2.68 D in the first year, 2.21 D in the second year, and 4.84 D in the third year. In comparison with the above-mentioned studies which have been performed in patients with progressive keratoconus, our results showed a milder effect from crosslinking. This discrepancy is probably related to the stage of the disease and corneal characteristics such as rigidity, CCT, and maximum K values. With gradual progression from early to advanced keratoconus, affected eyes demonstrate more significant clinical findings, decreased biomechanical stiffness, and structurally weakened tissue.^13^,^22^ Corneal rigidity in patients with early keratoconus is only slightly less than normal and crosslinking possibly induces little change in its structure, reflected as a smaller effect from crosslinking. In contrast, patients with progressive keratoconus have severe corneal changes and very weak corneas. Collagen crosslinking in these patients probably causes more significant changes in refractive error and corneal curvature. We believe that the efficacy of collagen crosslinking in early and advanced keratoconus may be similar; the greater clinical effect seen in progressive disease may be due to greater reduction in corneal rigidity.

In conclusion, we recommend collagen crosslinking for patients with early keratoconus who cannot be optically corrected and those who demonstrate recent progression. It may be preferable to delay such treatment for patients that are adequately corrected and show very slowly progressive or non-progressive disease. Further studies with larger sample size and longer follow-up are required to determine the optimal time for intervention and the long-term effects of crosslinking for early keratoconus.

## References

[1] Krachmer JH, Feder RS, Belin MW (1984). Keratoconus and related noninflammatory corneal thinning disorders. Surv Ophthalmol.

[2] Rabinowitz YS (1998). Keratoconus. Surv Ophthalmol.

[3] Sekundo W, Stevens JD (2001). Surgical treatment of keratoconus at the turn of the 20th century. J Refract Surg.

[4] Wollensak G, Spoerl E, Seiler T (2003). Riboflavin/ultraviolet-a-induced collagen crosslinking for the treatment of keratoconus. Am J Ophthalmol.

[5] Wollensak G, Spörl E, Reber F, Pillunat L, Funk R (2003). Corneal endothelial cytotoxicity of riboflavin/UVA treatment in vitro. Ophthalmic Res.

[6] Wollensak G, Spoerl E, Reber F, Seiler T (2004). Keratocyte cytotoxicity of riboflavin/UVA-treatment in vitro. Eye (Lond).

[7] Wollensak G, Spoerl E, Seiler T (2003). Stress-strain measurements of human and porcine corneas after riboflavin-ultraviolet-A-induced cross-linking. J Cataract Refract Surg.

[8] Wollensak G (2006). Crosslinking treatment of progressive keratoconus: new hope. Curr Opin Ophthalmol.

[9] Wollensak G, Aurich H, Pham DT, Wirbelauer C (2007). Hydration behavior of porcine cornea crosslinked with riboflavin and ultraviolet A. J Cataract Refract Surg.

[10] Spoerl E, Huhle M, Seiler T (1998). Induction of cross-links in corneal tissue. Exp Eye Res.

[11] Spoerl E, Seiler T (1999). Techniques for stiffening the cornea. J Refract Surg.

[12] Braun E, Kanellopoulos J, Pe L, Jankov M (2005). Riboflavin ultraviolet A-induced collagen cross-linking in the management of keratoconus. Invest Ophthalmol Vis Sci.

[13] Caporossi A, Baiocchi S, Mazzotta C, Traversi C, Caporossi T (2006). Parasurgical therapy for keratoconus by riboflavin-ultraviolet type A rays induced cross-linking of corneal collagen: preliminary refractive results in an Italian study. J Cataract Refract Surg.

[14] Chan CC, Sharma M, Wachler BS (2007). Effect of inferior-segment Intacs with and without C3-R on keratoconus. J Cataract Refract Surg.

[15] Spoerl E, Wollensak G, Dittert DD, Seiler T (2004). Thermomechanical behavior of collagen-cross-linked porcine cornea. Ophthalmologica.

[16] Wollensak G, Wilsch M, Spoerl E, Seiler T (2004). Collagen fiber diameter in the rabbit cornea after collagen crosslinking by riboflavin/UVA. Cornea.

[17] Spoerl E, Wollensak G, Seiler T (2004). Increased resistance of crosslinked cornea against enzymatic digestion. Curr Eye Res.

[18] Mazzotta C, Balestrazzi A, Traversi C, Baiocchi S, Caporossi T, Tommasi C (2007). Treatment of progressive keratoconus by riboflavin-UVA-induced cross-linking of corneal collagen: ultrastructural analysis by Heidelberg Retinal Tomograph II in vivo confocal microscopy in humans. Cornea.

[19] Hafezi F, Kanellopoulos J, Wiltfang R, Seiler T (2007). Corneal collagen crosslinking with riboflavin and ultraviolet A to treat induced keratectasia after laser in situ keratomileusis. J Cataract Refract Surg.

[20] Raiskup-Wolf F, Hoyer A, Spoerl E, Pillunat LE (2008). Collagen crosslinking with riboflavin and ultraviolet-A light in keratoconus: long-term results. J Cataract Refract Surg.

[21] Pinelli R In My Hands: Corneal collagen cross-linking with riboflavin (C3-R) treatment opens new frontiers for keratoconus and corneal ectasia. ASCRS Eyeworld.

[22] Andreassen TT, Simonsen AH, Oxlund H (1980). Biomechanical properties of keratoconus and normal corneas. Exp Eye Res.

